# Research progress on detection techniques for point-of-care testing of foodborne pathogens

**DOI:** 10.3389/fbioe.2022.958134

**Published:** 2022-08-08

**Authors:** Sha Liu, Kaixuan Zhao, Meiyuan Huang, Meimei Zeng, Yan Deng, Song Li, Hui Chen, Wen Li, Zhu Chen

**Affiliations:** ^1^ Hunan Key Laboratory of Biomedical Nanomaterials and Devices, Hunan University of Technology, Zhuzhou, China; ^2^ Zhuzhou Hospital Affiliated to Xiangya School of Medicine, Department of Pathology, Central South University, Zhuzhou, China; ^3^ College of Food Science and Engineering, Central South University of Forestry and Technology, Changsha, China

**Keywords:** foodborne pathogens, rapid detection, immunoassay, molecular biology, POCT, microfluidic chip, biosensor

## Abstract

The global burden of foodborne disease is enormous and foodborne pathogens are the leading cause of human illnesses. The detection of foodborne pathogenic bacteria has become a research hotspot in recent years. Rapid detection methods based on immunoassay, molecular biology, microfluidic chip, metabolism, biosensor, and mass spectrometry have developed rapidly and become the main methods for the detection of foodborne pathogens. This study reviewed a variety of rapid detection methods in recent years. The research advances are introduced based on the above technical methods for the rapid detection of foodborne pathogenic bacteria. The study also discusses the limitations of existing methods and their advantages and future development direction, to form an overall understanding of the detection methods, and for point-of-care testing (POCT) applications to accurately and rapidly diagnose and control diseases.

## Introduction

Foodborne pathogens continue to cause many intestinal diseases in humans around the world, causing a huge health and economic burden (Ling et al., 2019; Akter et al., 2021; Prata et al., 2021; Qiu et al., 2021). Figures from the World Health Organization (WHO) estimate that about 2 billion people die each year from diarrhea or disease caused by contaminated food, and 30% of them are children under 5 years of age (Scallan Walter et al., 2020; Belina et al., 2021; Van Puyvelde et al., 2021). The United States has one of the safest food supplies in the world, yet one in four people gets sick from foodborne diseases every year (Hoffmann and Walter, 2020; Hoffmann et al., 2021; Ge et al., 2022). The frequency and importance of these foodborne diseases depend on interactions between foodborne pathogens, hosts, food, and the environment (Ishaq et al., 2021; Jahan et al., 2021; Saravanan et al., 2021; Zarkani and Schikora, 2021). Bacterial foodborne illnesses are caused by infections with bacterium, such as *Salmonella, Campylobacter* spp., *Escherichia coli, Shigella, Vibrio, Listeria monocytogenes* (LM) and *Clostridium botulinum*, and *Clostridium perfringens* (Liu et al., 2019a; Xiao et al., 2019; Christopher et al., 2021; Fuochi et al., 2021; Kim et al., 2021a; Darbandi et al., 2022; Oyejobi et al., 2022). Viruses commonly reported are Norovirus and Hepatitis A, while examples of parasites involved are *Cryptosporidium* spp, *Giardia lamblia*, *Trichinella spiralis*, *Cyclospora* spp, *Toxoplasma canis,* and *Entamoeba histolytica* (Stryinski et al., 2020; Bozkurt et al., 2021; Lee and Yoon, 2021; Segeritz et al., 2021; Patel et al., 2022). Typical symptoms of foodborne illness include abdominal pain, diarrhea, vomiting, nausea, fever, difficulty breathing, and even death in severe cases (Abebe et al., 2020; Aik et al., 2020; Myintzaw et al., 2021; Sun et al., 2021; Janekrongtham et al., 2022). These symptoms are caused by ingested pathogens, such as foodborne infections (*Salmonellosis*, *Listeriosis*, etc.) (Gallo et al., 2020; Jang et al., 2021), or by microbial toxins produced in the host, such as toxic infections (*C*. *perfringens* food poisoning, etc.) (Rajkovic et al., 2020; Sharma et al., 2021). In the case of foodborne poisoning, toxins produced by pathogens in food cause symptoms (*C*. *botulinum* food poisoning, etc.) (Augustin et al., 2020; Walter et al., 2021). Poultry, ground meat, seafood, milk and dairy products, fruits, and vegetables have been blamed for most of the outbreaks (Leon Madrazo and Segura Campos, 2020; Visciano and Schirone, 2021; Singha et al., 2022).

Because foodborne pathogens pose a great threat to public health, it is therefore important to detect these pathogens (Dumen et al., 2020; Teffo and Tabit, 2020; Du et al., 2021a; Mi et al., 2021). Traditional methods for food pathogen detection mainly include plate separation, chemical analysis, and immunoassay (Wang et al., 2020a; Wang et al., 2020b; Han et al., 2021; Weng et al., 2021). However, these methods have more or less shortcomings, such as cumbersome steps, long detection cycle, high cost, and high requirements for a professional level of operators (Zhang et al., 2020a; Vidyadharani et al., 2021; Xie et al., 2021a; Nassarawa et al., 2022). Therefore, it is urgent to develop simple, sensitive, rapid, and low-cost methods for the detection of pathogenic bacteria in complex food samples.

**FIGURE 1:**
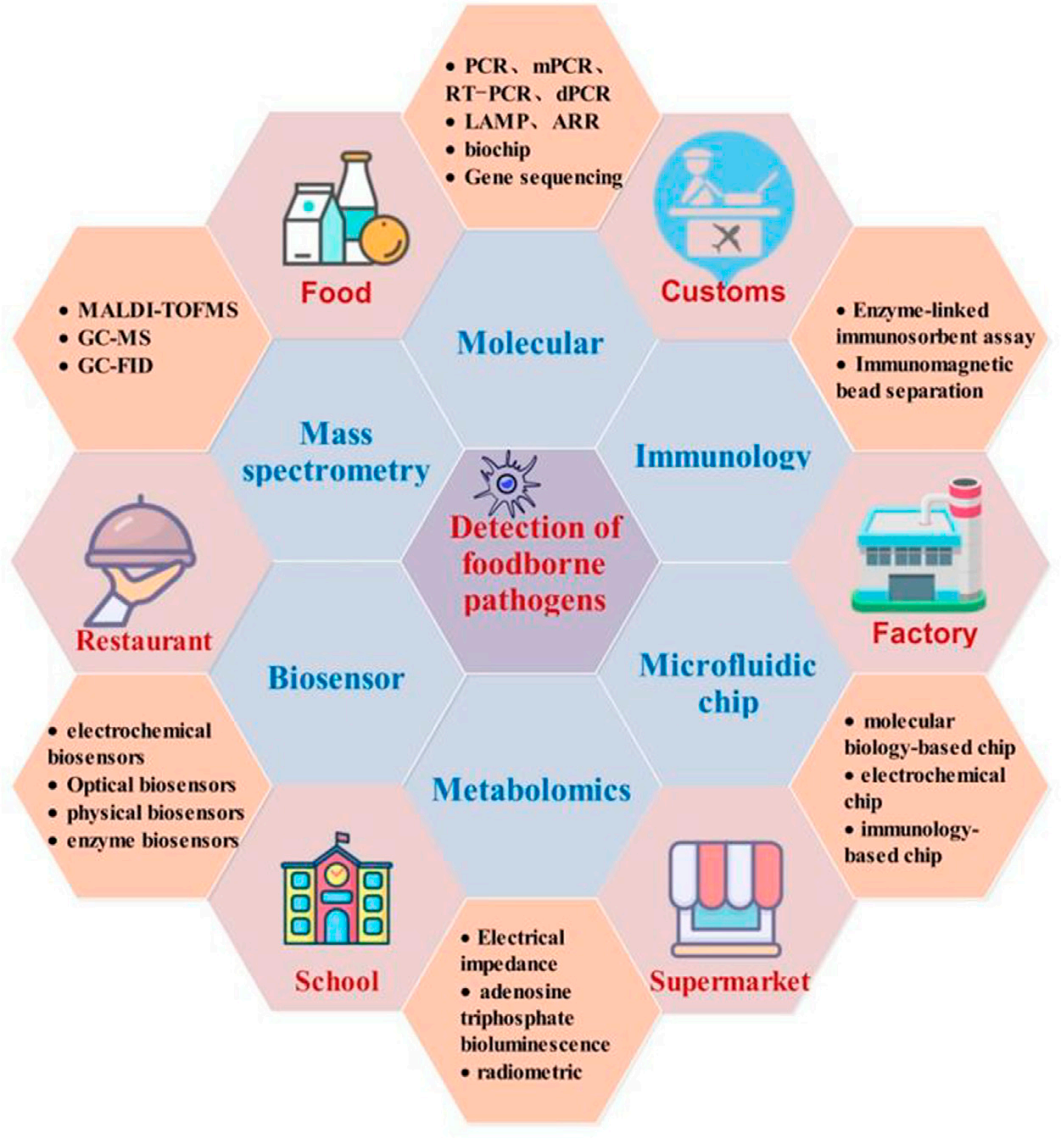
Summary based on detection methods of foodborne pathogenic bacteria.

Point-of-care testing (POCT) technology is a rapidly developing foodborne pathogen detection method in recent years that has advantages, such as simple operation, rapid operation, portability, and automation (Huang et al., 2018; Xu et al., 2021). In this study, applications of POCT in the detection of foodborne pathogens based on biomolecules, immunoassay, gene sequencing, microfluidic, metabolism, biosensor, mass spectrometry, and related technologies in recent years have been reviewed (Figure 1). The principle, advantages, and disadvantages of each method and its application status are described. The existing problems and future development of rapid detection methods are also discussed. This study provides a reference for the development of rapid detection technology for foodborne pathogens and has certain significance for research on various disciplines and food safety supervision.

## Immunological detection techniques

From its birth to its current development, the wide application of immunological detection technology determines its dominant position in the fields of biological science, food science, and clinical medicine. Its principle is based on antibody–antigen interaction, that is, the binding of specific antibodies (polyclonal antibody or monoclonal antibody) to their specific antigens (Jayan et al., 2020; Mishra et al., 2020; Wang et al., 2020c; Pires et al., 2021; Sohrabi et al., 2022).

### Enzyme-linked immunosorbent assay

Enzyme-linked immunosorbent assay (ELISA) is a sensitive and specific analytical biochemical method that can be used for the detection and quantitative or qualitative analysis of analytes without requiring sophisticated or expensive equipment (Ferone et al., 2020; Nath et al., 2020; Rani et al., 2021; Kotsiri et al., 2022). At present, although traditional ELISA is widely used in scientific research and testing institutions and is the ideal method for the detection of viruses and antibodies (Leva-Bueno et al., 2020; Navarro et al., 2020; Xiao et al., 2021), this traditional method is time-consuming and also requires skilled operation techniques and sophisticated instruments (Luo et al., 2020a; Razmi et al., 2020; Huang et al., 2022). So the researchers changed the traditional approach.

**FIGURE 2:**
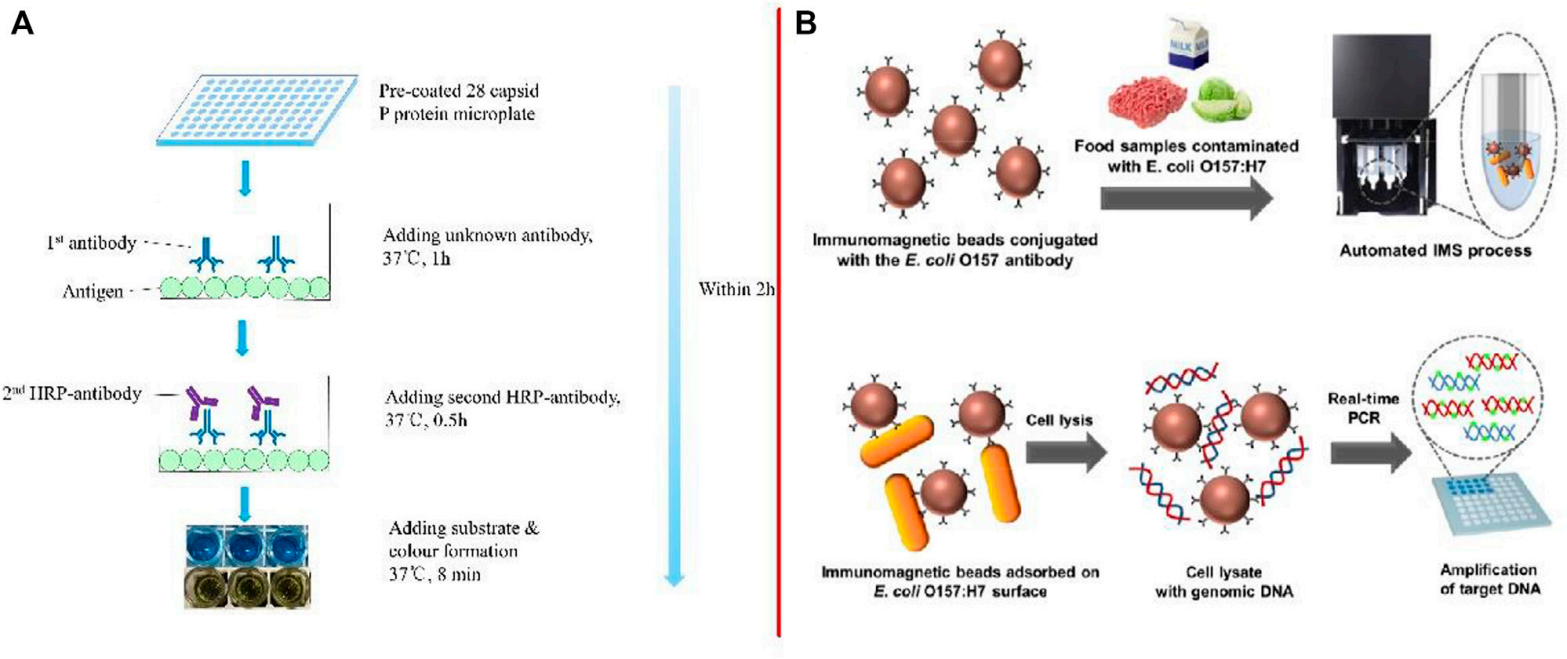
**(A)** Step-by-step schematic representation of ELISA (Zuo et al., 2021); **(B)** Schematic illustration of the detection method for *E. coli* O157:H7 using the automated IMS device combined with real-time PCR (Park et al., 2020).

Zuo et al. (2021) developed an indirect ELISA for testing the broad spectrum of anti-NoV antibodies (Figure 2A). The entire process of testing the spectrum of unknown antibodies required 2 h for completion. The intra-assay and inter-assay coefficients of variation were less than 10%. He et al. (2020) developed a sandwich-ELISA, which could detect 6 CFU/ml of *Salmonella enteritidis* (*S. enteritidis*) in milk after 10 h of enrichment. Zhao et al. (2020) developed a wax-printed paper-based enzyme-linked immunosorbent assay (P-ELISA) based on microfluidic paper-based analytical devices, with the whole operation time being less than 3 h and only needing 5 μl of samples for detection. The limit of detection for *E*. *coli* O157:H7 (*E. coli* O157:H7) reached 10^4^ CFU/ml.

### Immunomagnetic separation technology

Immunomagnetic separation (IMS) is an effective pre-concentration technique for food samples that can quickly and selectively separate and concentrate target bacteria from complex food substrates (Liu et al., 2019c; Fang et al., 2021a; Nadar et al., 2021). The main principle is the surface of superparamagnetic particles after chemical modification, combined with target bacteria-specific active protein made of immunomagnetic bead separation (IMBS), and then the antibodies on IMBS will specifically identify and capture the target bacteria in the samples to be tested, which leads to the formation of IMBS-target bacteria complex (Ma et al., 2012; Jiang et al., 2013; Shen et al., 2021a; Zhang et al., 2021a). Finally, the complex is rapidly separated from other impurities in the sample by the force of a magnetic field, so as to achieve the efficient and accurate concentration of the target microorganism (Li et al., 2015; Wang and Lin, 2020; Wang et al., 2020d; Hou et al., 2020; Tang et al., 2020).

IMS technology has the characteristics such as strong specificity, high sensitivity, and fast separation speed (Pissuwan et al., 2020; Zhang et al., 2020b; Qi et al., 2022) and can be combined with a variety of other technologies (Yao et al., 2020; Zhai et al., 2021; Zhao et al., 2021a; Dester and Alocilja, 2022), such as ELISA, chemiluminescence immunoassay (CLIA), flow cytometry (FCM), immunochromatography (ICA), polymerase chain reaction (PCR), and other detection methods, to make the detection process more rapid and efficient (Yang et al., 2014a; He et al., 2017; Li et al., 2017; Lin et al., 2020; Nguyen and Kim, 2020; Sourri et al., 2022).

Moreover, Park et al. (2020) described the development of an automated IMS device combined with real-time PCR for detecting foodborne bacteria (Figure 2B). Target bacteria in the range of 10^1^–10^2^ colony-forming units per mg or g of sample can be detected in food samples, such as milk, ground beef, and cabbage, by using the proposed approach. Lv et al. (2021) developed an IMS technique by combining improved propidium monoazide and droplet digital PCR to detect the pathogenic viable but non-culturable *Cronobacter sakazakii* (*C. sakazakii*). The detection limit for this method in a background of powdered infant formula (PIF) was 5.6 copies/g. Jiang et al. (2020) first detected *Vibrio parahaemolyticus* (*V. parahaemolyticus*) in oysters by recombinant enzyme polymerase amplification (RPA) and side-flow (LF) combined with IMS. The method effectively combined sample preparation, amplification, and detection on one platform and could also detect *V. parahaemolyticus* within 15 min.

The IMS technology also has some limitations, such as the selected antigen target should have strong specificity, can specifically enrich the target bacteria, and avoid the enrichment of miscellaneous bacteria (Wang et al., 2020e; Zhai et al., 2020). Therefore, in order to rapidly develop in the field of foodborne pathogenic bacteria detection, specific antibodies from pathogenic bacteria must be screened.

### Other immunological techniques

In addition to the above two immunological detection techniques, immuno chromatography (IC), immunodiffusion, immunofluorescence, Western blot, and Latex agglutination have all been applied in the detection of foodborne pathogens (Zhou et al., 2017; Li et al., 2018; Wu et al., 2020a; Morales-Pablos et al., 2020; Gao et al., 2021; Lopes-Luz et al., 2021; Wangman et al., 2021; Zhao et al., 2021b). Immunology technology has good specificity, high efficiency, low testing cost, and does not need the advantages of large instrument, but when the influenza virus contains competitive target bacteria in food material is very likely a false-positive result, the sensitivity is not high, these factors limit the immunology technology widely application in detection of foodborne pathogenic bacteria (Wang and Park, 2020; Zhao and Wu, 2020; Yan et al., 2021).

## Molecular biology detection technology

Molecular biology detection technology is based on nucleic acid, through the detection of specific target pathogens DNA or RNA for detection purposes (Xi et al., 2014; Liu et al., 2017a; Foddai and Grant, 2020; Zheng and Tan, 2020; Kim and Oh, 2021). It is achieved by hybridizing the target sequence with complementary probes or primers (Yang et al., 2014b; Yang et al., 2017; Chen et al., 2020a; Wachiralurpan et al., 2020).

### Temperature-changing amplification technology

Variable temperature amplification is a method based on PCR technology. Conventional PCR can only detect one pathogen at a time, but there are many pathogenic bacteria in food (Kim and Kim, 2021). Therefore, based on conventional PCR, dozens of different types of PCR methods were derived, mainly including multiple polymerase chain reaction (mPCR), real-time quantitative polymerase chain reaction (qPCR), and digital polymerase chain reaction technology (dPCR) (Tang et al., 2018; Mou et al., 2019a; Lei et al., 2020a; Cardoso et al., 2020; Huang et al., 2021a).

mPCR is based on traditional PCR, whereby multiple pairs of specific primers and templates are added into the same PCR reaction system (primers are specifically bound to corresponding templates) to amplify multiple DNA fragments with different sequences (Zhang et al., 2020c; Hossain et al., 2021; Bonny et al., 2022; Liu et al., 2022). Multiple DNA fragments amplified in the same reaction system can simultaneously detect multiple foodborne pathogens, reduce the number of experimental operations, shorten the detection time, and save reagents (Ma et al., 2020a; Yang et al., 2020a). He et al. (2022) developed a detection system based on magnetic separation, mPCR, and capillary electrophoresis (CE) technologies for the simultaneous detection of four foodborne pathogens. The detection limit for bacterial DNA reached 10^−5^–10^−7^ ng/μl and in the analysis of mocked food samples, the assay showed good sensitivity for bacterial detection ranging from 10^1^ to 10^5^ CFU/ml with excellent specificity.

mPCR is suitable for the detection of multiple foodborne pathogens with the same symptoms or easily contaminating the same food, which can standardize the detection of microorganisms in food (Du et al., 2020a; Ripolles-Avila et al., 2020). However, because multiple pairs of primers are amplified in the same system, each pair of primers affects each other, so the amplification effect and the actual number of amplified fragments in the actual operation of mPCR are often not satisfactory (Luo et al., 2020; Chen et al., 2021).

**FIGURE 3:**
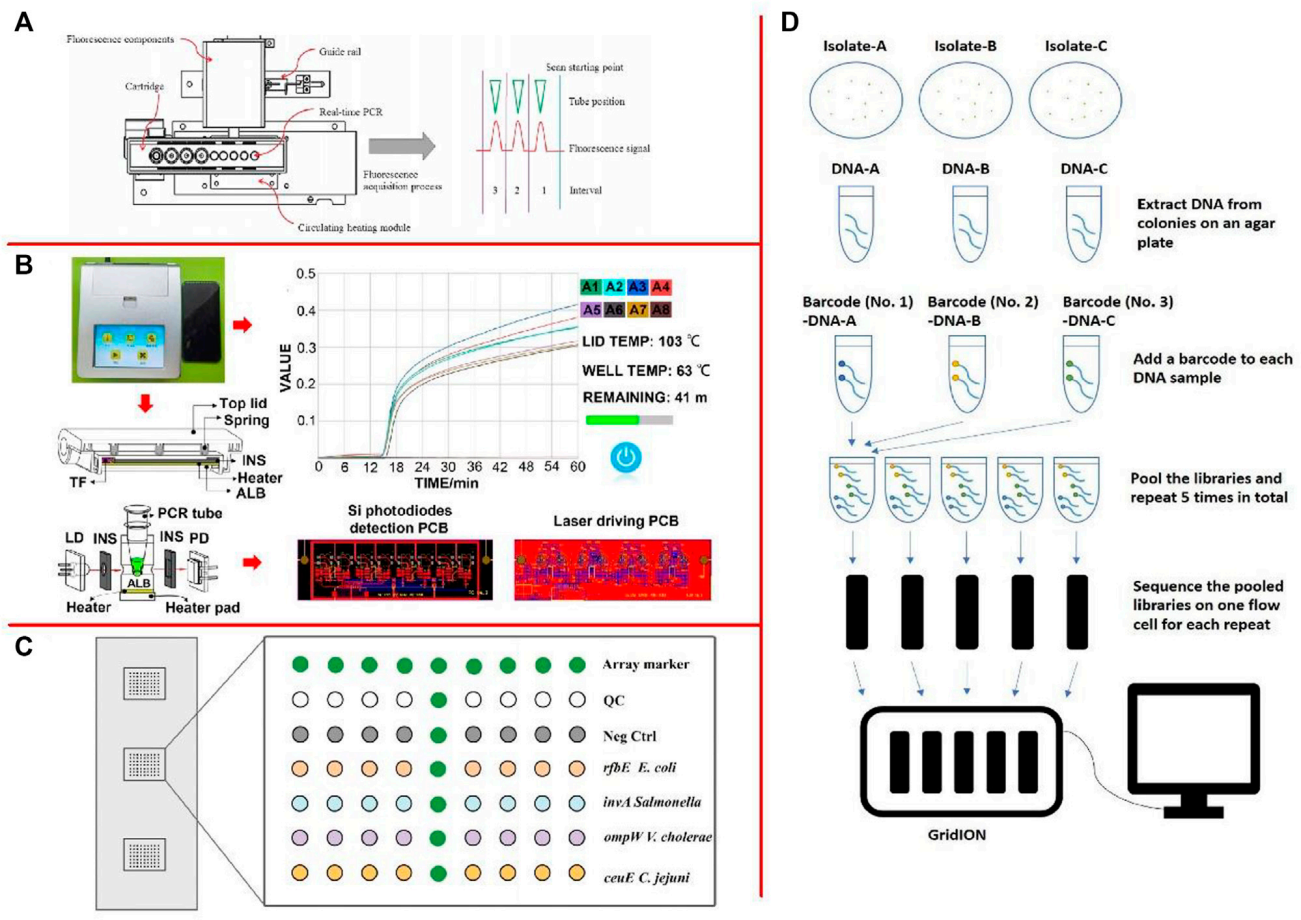
**(A)** Positions of the fluorescence detection module and the Peltier device, and the principle of fluorescence acquisition (Chen et al., 2017); **(B)** The design of the diagnostic device (Chen et al., 2022); **(C)** Layout of oligonucleotide probes on the detection microarray (Liu et al., 2017b); **(D)** An example of the workflow of multiplex ONT sequencing library preparation and sequencing (Wu et al., 2021).

qPCR operates by adding fluorescent-labeled probes or fluorescent substances into the PCR system and monitors the accumulation of fluorescence signals in the whole PCR amplification process in real-time through the instrument. Finally, the method for quantitative analysis of samples with unknown concentrations was carried out through standard curves (Fu et al., 2020; Baoutina and Bhat, 2021; Wan et al., 2021). Moreover, Chen et al. (2017) presented a new POCT system based on magnetic nanoparticles that enable sample in-answer out (SIAO) automated real-time testing for pathogens (Figure 3A). Real-time PCR by the two methods (TaqMan-based probe and SYBR green dye) in the SIAO system was achievable by the manual method with comparable results. Xie and Liu. (2021) developed a double TaqMan real-time fluorescence quantitative PCR (DRT-PCR) method for their simultaneous enumeration within two kinds of powdered infant foods (PIFs). The dRT-PCR could quantify as low as 10^2^ and 10^1^ CFU/ml *C. sakazakii* and *Staphylococcus aureus* (*S. aureus*) in both pure cultures and spiked PIFs.

Compared with ordinary PCR, the nucleic acid amplification of qPCR technology is completed in a closed system, and no electrophoresis analysis is required after the amplification, which not only reduces the chances for sample contamination, but also avoids false-positive results caused by contamination and also shortens the detection time (Obande and Singh, 2020; Kim et al., 2021b). However, the experimental cost of real-time quantitative PCR is high, and the equipment required is expensive, which also requires the operator to have a high level of professional technology (Nunez-Bajo et al., 2020; Wang et al., 2021a).

dPCR does not require the establishment of a standard curve and concentration comparison by Ct value (the number of cycles that PCR fluorescence signal goes through when it reaches a set threshold) and is considered an absolute quantitative method (Grudlewska-Buda et al., 2020; Lei et al., 2021; Plante et al., 2021). Droplet digital PCR (ddPCR) is a new method that disperses a single target DNA molecule into multiple separated droplets, detects each droplet one by one after PCR amplification, and accurately quantifies DNA copy number (Lei et al., 2020b; Iwu et al., 2020). Du et al. (2021b) developed an effective pretreatment method based on an *in-situ* enrichment culture with an immunomagnetic separation step, combined with ddPCR technology to achieve rapid detection of trace *Salmonella* in milk, which allowed detecting as low as 10^−1^ CFU/ml level of *Salmonella*. ddPCR has the advantages of high sensitivity, high accuracy, high tolerance, and absolute quantification and has been widely used in rare mutation detection and gene expression detection in complex samples (Salipante and Jerome, 2020; Yang et al., 2020b).

### Constant temperature amplification technology

At present, constant temperature amplification techniques applied to foodborne pathogenic microorganisms mainly include loop-mediated isothermal amplification (LAMP), recombinant enzyme-mediated nucleic acid amplification (RAA), RPA, and nucleic acid sequence-dependent amplification (NASBA) (Chen et al., 2018; Khan et al., 2019; Safenkova et al., 2020; He et al., 2021a; Hoang et al., 2021; Tian et al., 2022).

LAMP is a mature isothermal nucleic acid amplification technique in which the target sequence was amplified with two or three sets of primers at a constant temperature of 60–65°C (Prasannakumar et al., 2020; Wang et al., 2020f; Zhang et al., 2021b). Typically, four different primers are used to amplify six different regions on the target gene, which increases specificity (Xie et al., 2022). Another pair of “cyclic primers” can further accelerate the reaction. In addition to replication activity, polymerases with high chain displacement activity are required for amplification (Garafutdinov et al., 2020; Padzil et al., 2022). Liu et al. (2022) developed a LAMP method for LM detection using SYTO9 staining and image processing techniques. The detection limit of LM was 6 copies/μl. Chen et al. (2022) developed a novel Enter cytozoon hepatopenaei (EHP) field rapid detection device (size 18.8 × 16.7 × 6.6 cm^3^) based on magnesium pyrophosphate precipitation and LAMP (Figure 3B). The detection limit for EHP was 0.1 fg/μl. Moreover, Jin et al. (2020) developed a LAMP-based microdevice for performing high-throughput visual detection. The approach was able to perform simultaneous identification of six foodborne pathogens within 1 h. LAMP is an isothermal amplification technique with high practical value and detection efficiency, but it also has obvious disadvantages, such as false-positive results after the addition of ring primers (Yu et al., 2021a). Although the frequency of false-positive can be reduced by various methods, it is still impossible to avoid the high requirement and difficulty in primer design.

RAA is a technique for nucleic acid amplification using recombinant enzyme, single-chain binding protein, and DNA polymerase under isothermal conditions (optimal temperature 37°C) (Feng et al., 2022a; Hou et al., 2022; Zhang et al., 2022). The established RAA method can effectively shorten the detection time and does not require temperature change during nucleic acid amplification (Aman et al., 2020; Teklemariam et al., 2020; Li et al., 2021a). Li et al. (2021b) introduced the transcleavage activity of CRISPR/Cas12a into an electrochemical biosensor (ECRISPR), combined with RAA, to establish a cost-effective, specific, and ultrasensitive method, namely, RAA-based E-CRISPR. Under optimized conditions, the RAA-based E-CRISPR can detect as low as 0.68 aM of genomic DNA and 26 CFU/ml of LM in pure cultures. Xie et al. (2021b) proposed a modified propidium monoazide (PMAxx) dye combined with RAA for the rapid and real-time detection of viable *S. aureus*. The detection limit for viable *S. aureus* was 10^2^ CFU/ml under 3 h enrichment and 10^1^ CFU/ml under 6 h enrichment in artificially contaminated milk, respectively. Zhou et al. (2022) reported a novel CRISPR/Cas12a-based fluorescence enhanced lateral flow biosensor (LFB) in conjunction with functionalized quantum dots, combined with RAA, to establish low-cost, simple, and sensitive detection of *S. aureus*, namely, CRISPR/Cas-recombinase-assisted amplification-based LFB (CRA-LFB). The limit of detection was as low as 75 aM of genomic DNA, and 5.4 × 10^2^ CFU/ml of *S. aureus* in pure cultures were detected. RAA has a great development advantage due to its lower requirements on environmental temperature, operating skills, and experimental equipment (Mu et al., 2021).

### Biochip technology

Biochip technology was started in the 1980s. It is a micro biochemical analysis system of molecular microarray. It uses mechanical arm sampling technology or microelectronic lithography technology to construct up to tens of thousands of different probes on the surface of a certain volume of the solid carrier to detect a variety of biological components (Li et al., 2013; Azizipour et al., 2020; Aladese and Jeong, 2021; Tahir et al., 2021). The biochip technology has the advantages such as diversification, high throughput, short detection time, and portability. At present, gene chip, protein chip technology, and liquid chip are widely used in the detection of foodborne pathogens (Pos et al., 2020; Qian et al., 2022).

Gene chip is the first developed and earliest researched and developed technology in biochip technology (Zeng et al., 2014; Chen et al., 2020b; Kumar et al., 2020; Ali et al., 2021). The sequencing principle for the gene chip is the hybridization sequencing method, by which hybridization with a group of nucleic acid probes with a known sequence of target nucleotide, and with a known sequence for nucleic acid sequencing are fixed on the surface of a substrate, (Zhang et al., 2020d; Hariharan and Prasannath, 2021; Taguchi et al., 2021). Liu et al. (2017b) developed a magnetic nanoparticle-enhanced oligonucleotide microarray assay for rapid and sensitive identification of *E. coli* O157:H7, *Salmonella enterica*, *Vibrio cholerae*, and *Campylobacter jejuni* (*C. jejuni*) in food (Figure 3C). In comparison with conventional single-stranded target preparation methods, this magnetic nanoparticles-based method yielded up to 15-fold increase in the hybridization signal and achieved 1 similar to 2 orders of magnitude enhancement on the limit of detection. Sarengaowa et al. (2020) developed an in situ-synthesized gene chip for the detection of foodborne pathogens on fresh-cut fruits and vegetables. The detection limit for the five target pathogens on fresh-cut cantaloupe and lettuce was approximately 3 log CFU/g without culturing and with a detection time of 24 h. Shen et al. (2020) performed the genome-wide DNA microarray analysis using *S*. *typhimurium* incubated with 0.001% epsilonpolylysine in 0.1% Bacto Soytone at 30°C for 2 h.

The high degree of automation of gene chip technology can analyze a large number of samples at one time, and the data are objective and reliable (Jia et al., 2021a). But the cost is high, with low detection sensitivity, poor repeatability, and narrow analysis scope (Zaczek-Moczydlowska et al., 2021).

Protein chip technology is a kind of protein microarray, which is different from gene chip to realize binding based on the principle of base complementary pairing. It uses the interaction between proteins, such as the reaction between antigen and antibody, enzyme and substrate, for detection (Khan et al., 2021; Zhou et al., 2021; Hang et al., 2022). With the continuous development and improved protein microarray technology, the technology has been gradually applied to the detection of foodborne pathogens. Liu et al. (2019c) constructed a protein chip to screen for antibody titers present in test sera raised against whole *C. jejuni* cells with over 1,400 individually purified GST-tagged *C. jejuni* proteins, representing over 86% of the proteome. These results indicated that the unbiased chip-based screen can reveal the full repertoire of host antibodies against microbial proteomes. Protein microarray technology is an emerging technology with bright development prospects, but there are still some problems in maintaining protein activity, protein fixation methods, and detection sensitivity (Xia et al., 2021), which need further research and optimization.

Liquid chips, also known as microsphere suspension chips, began in the mid-1990s, with their diverse fluorescent encoded microspheres, up to 100 different probes can be crosslinked by different ways of binding and hybridization (Jia et al., 2021b). Qualitative and quantitative detection of microsphere fluorescence coding and molecular fluorescence intensity by two different laser beams is a new generation of high-throughput molecular detection technology platform following DNA chip and protein microarray (Han et al., 2020; Li et al., 2020; Yin et al., 2020). Pang et al. (2019) established a microsphere-based suspension array (MSA) for the detection and identification of 55 *V. parahaemolyticus* K-serogroups based on CPSgc-specific genes. This system was then used to examine 845 publicly available *V. parahaemolyticus* genomes. Shi et al. (2019) established a rapid and accurate method based on mPCR combined with suspension array flexible sequence-tagged (xTAG) technology to simultaneously detect *S*. *typhimurium*, *Brucella* spp., *Bacillus* cereus, and *Shigella* spp. in raw milk. The results showed that the detection of milk samples demonstrated 100% specificity.

### Gene sequencing

In molecular biology, DNA sequence analysis is the basis for further research and modification of target genes (Mou et al., 2019b; Uelze et al., 2020; Hu et al., 2021). At present, there are two types of mainstream technologies for sequencing: 1) Traditional sequencing technology, or first-generation sequencing technology, is represented by the Sanger sequencing method (Segerman, 2020; Kaprou et al., 2021). 2) The next-generation sequencing (NGS) technology developed in recent years is also known as high-throughput sequencing technology, and its sequencing principles include simultaneous sequencing and single-molecule sequencing (Reuter et al., 2015; Lu et al., 2020; Van Poelvoorde et al., 2020). Specifically, synthetization sequencing (also known as the second-generation sequencing technology) is represented by Roche’s 454 technology, Illumina’s Solexa, Hiseq technology, and ABI’s Solid technology (Lewis et al., 2020; Shen et al., 2021b). Single-molecule sequencing (also known as third-generation sequencing technology) is based on Helicos Bioscience’s HeliScope genetic analysis system, Pacific Biosciences’ PacBio RS single-molecule real-time sequencing system, and Oxfold Nanopore Examples include Technologies’ GridION and MinION (Yu et al., 2020; Gunther et al., 2021; Van Reckem et al., 2021). Single-cell sequencing technology is to sequence each individual cell through high-throughput sequencing technology to obtain the genetic information of each individual cell (Davey and Valdivia, 2020; Peng et al., 2020a).

The principle for the Sanger sequencing method is to randomly cut genomic DNA into small fragments, and then many small fragments of DNA are cloned into plasmid vectors and transformed into *E*. *coli*. Finally, the cultured *E*. *coli* extracts plasmid for sequencing, and each sequencing reaction is completed in a reaction system for only a few microliters (Efimochkina and Sheveleva, 2022). Syromyatnikov et al. (2020) used high-throughput sequencing and Sanger sequencing of individual bacterial colonies to analyze the microbial content of commercially available butter brands. We identified a total of 94 amplicon sequence variants corresponding to different microbial taxa. Sanger sequencing technology is relatively common in small bacterial genome sequencing, plasmid sequencing, and other research fields, with its accuracy, precision target, and low throughput (Maguire et al., 2021). However, in large-scale sequencing tasks, Sanger sequencing technology has defects of low throughput, slow speed, and high cost, thus promoting high-throughput sequencing technology (Sheka et al., 2021).

High-throughput sequencing technology is a revolutionary improvement on traditional sequencing technology in history, which can simultaneously determine the molecular sequence of millions or even tens of millions of DNA (Cassotta et al., 2020; Chelliah et al., 2022). Muriuki et al. (2021) used 454 pyrosequencing, Illumina high-throughput sequencing of 16S rRNA gene in the analysis of total community DNA extracted from samples using the phenol-chloroform method. Uncultured *Candidatus* Koribacter and *Candidatus Solibacter* were also detected in the food samples. There was a significant difference in the microbial community structure among the sample types (*p* < 0.1). Gutierrez et al. (2021) sequenced 62 cases of Shiga toxin-producing *E*. *coli* (STEC) isolated from Chile using MiSeq Illumina. The results indicated that there may be local emerging STEC with unique features, nevertheless, no molecular markers were detected. Wu et al. (2021) evaluated the serotype prediction accuracy of using whole-genome sequencing (WGS) data from multiplex ONT sequencing (Figure 3D). This study demonstrated that accurate serotype prediction results could be obtained when multiplexing five or less *Salmonella* isolates with an average of 6 h of multiplex ONT sequencing, where each multiplexed isolate received at least 50× depth of genome coverage of sequencing data after demultiplexing.

The advantage of second-generation sequencing technology is that the cost is greatly reduced and the flux is greatly improved compared with the first generation, but the disadvantage is that the PCR process introduced will increase the sequencing error rate to a certain extent, and has a systematic bias, and the read length is relatively short (Kaavya et al., 2021). The third-generation sequencing technology is developed to solve the shortcomings of the second generation. Its fundamental feature is single-molecule sequencing, which does not require any PCR process, in order to effectively avoid system errors caused by PCR bias, while improving the read length, and maintaining the advantages of the second-generation technology of high throughput and low cost (Quijada et al., 2020).

## Microfluidic detection technology

Microfluidics provides a powerful tool for testing applications with its advantages of portability, miniaturization, automation, multi-channel sample testing, minimization of hazardous material handling, and cost savings (Ragab and El-Kimary, 2021; Su et al., 2021; Tseng et al., 2021). In addition, all analytical processes, including sample preparation, reaction, separation, and detection are integrated into a microfluidic chip for field test applications (Fu et al., 2021; Xie et al., 2021c). Biosensors that use a variety of technologies combined with microfluidic chips to detect foodborne pathogens have been widely reported (Ali et al., 2020; Kaya et al., 2021). Many new microfluidic chips have been successfully developed for the detection of foodborne pathogens. At present, according to the detection principle, the microfluidic detection chips are mainly divided into three categories: molecular biology-based microfluidic detection chips, immunology-based microfluidic detection chips, and electrochemical microfluidic detection chips.

**FIGURE 4:**
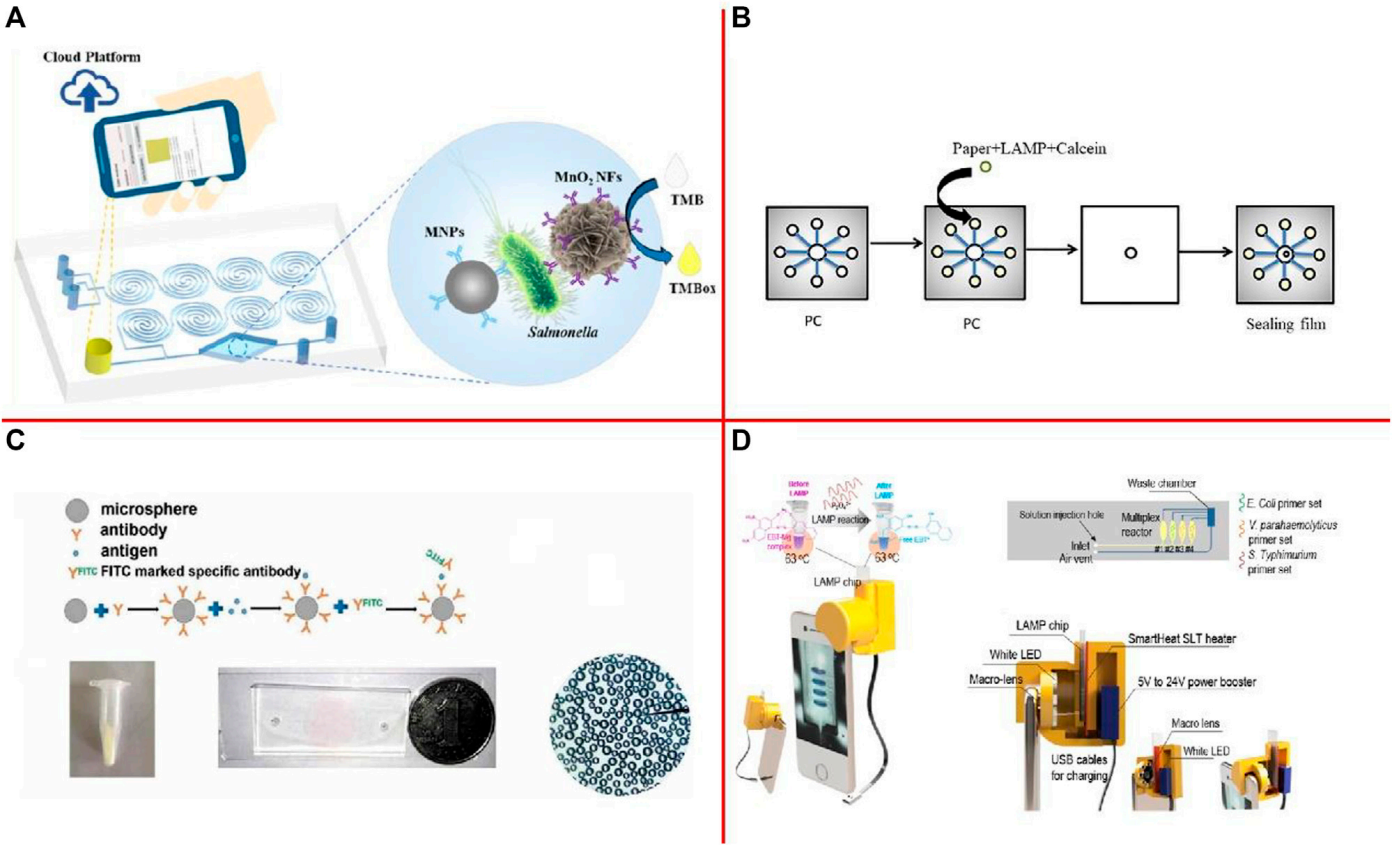
**(A)** Schematic of this Colorimetric Biosensor for Detection of *Salmonella* (Xue et al., 2021); **(B)** Making the process of the microchip (Zhang et al., 2021b); **(C)** The whole process of a microfluidic platform for detection of *S. aureus* by fluorescence labeling (Song et al., 2020); **(D)** Schematics of the i-Gene (Nguyen et al., 2020).

Xue et al. (2021) developed a microfluidic biosensor for rapid and sensitive detection of *Salmonella* using manganese dioxide nanoflowers (MnO_2_ NFs), a microfluidic chip with a convergence-divergence spiral micromixer, and a smartphone app with a saturation calculation algorithm (Figure 4A). This biosensor was able to detect *Salmonella* from 4.4 × 10^1^ to 4.4 × 10^6^ CFU/ml in 45 min with a detection limit of 44 CFU/ml. Zhang et al. (2021b) have created an embedded paper-based microchip based on LAMP which can rapidly and sensitively detect foodborne pathogens (Figure 4B). The detection limit for *Salmonella* spp. in the sample measured by the microchip was approximately 12 CFU/ml. Song et al. (2020) developed a microfluidic platform to detect *S. aureus* by fluorescence labeling method and a self-made microfluidic chip, which has immune spheres were used to study the effect of capturing *S. aureus* (Figure 4C). Results showed that our platform can detect *S. aureus* at an injection rate of 5 μl/min reacted for 4 min and the detection limit of bacteria was 1.5 × 10^1^ CFU/mul. Nguyen et al. (2020) proposed an integrated smartphone-based genetic analyzer. The LAMP mixture for Eriochrome Black T (EBT) colorimetric detection was injected into the LAMP chip to identify the *E. coli* O157:H7 (Figure 4D). The limit-of-detection (LOD) reached up to 10^1^ copies/μl. Moreover, Asgari et al. (2022) developed a sensitive Surface-enhanced Raman spectroscopy (SERS)-based microfluidic simmunosensor to separate and detect *E. coli* O157:H7 in romaine lettuce. The limit of detection for *E. coli* O157:H7 in romaine lettuce was found to be 0.5 CFU/ml. Wang et al. (2022) demonstrated an ultrasensitive and simple microfluidic immunosensor for a point-of-care test of *S. aureus* based on stir bar enrichment and DNAzyme-assisted click reaction. The detection limit was 3 CFU/ml. Jiang et al. (2021) designed a thread-based microfluidic electrochemical aptasensor, fabricated and tested by using label-free aptamer immunosensing technology for rapid and highly sensitive detection of *V. parahaemolyticus* in seafood. The proposed aptasensor has a dynamic detection range of 10–10^6^ CFU/ml, with a detection limit of 5.74 CFU/ml.

At present, microfluidic technology has shown great potential in environmental monitoring, food science, drug screening, disease diagnosis, and other fields, but there is still a long way to go to the market application. Therefore, the development of new materials and new processing methods is an important means to promote the development of microfluidic technology (Tsougeni et al., 2020).

## Metabolic detection techniques

Metabolic detection is a common technique for the detection of foodborne pathogens (Castle et al., 2021). Its principle is to use various techniques to detect the variation characteristics of the amount and type of primary metabolites or secondary metabolites produced by different pathogenic bacteria in a specific cultural environment, to identify the pathogenic bacteria (Duarte-Sierra et al., 2020; Subjakova et al., 2021). According to different detection technologies, it can be divided into electrical impedance technology, radiometric technology adenosine triphosphate (ATP) bioluminescence technology, microcalorimeter technology, etc. (Mobed et al., 2020; McCuskey et al., 2022).

### Electrical impedance technology

According to different metabolic activities of microorganisms in the growth process, electrical impedance technology is used to detect and identify microorganisms. Wang et al. (2020g) developed a sensitive electrochemical aptasensor using aptamer coated gold interdigitated microelectrode for targeted capture and impedance measurement, and antibody-modified nickel nanowires (NiNWs) for target separation and impedance amplification. This electrochemical aptasensor was able to quantitatively detect *Salmonella* ranging from 10^2^ to 10^6^ CFU/ml in 2 h, with a detection limit of 80 CFU/ml. Wang et al. (2021b) developed a novel impedance immunosensor based on a metal-organic framework (Mn-MOF-74) to rapidly and sensitively detect LM in milk. The recoveries for L.m cells at concentration between 1.0 × 10^0^ and 1.0 × 10^4^ CFU/ml were 90.2%–101.7% in water and 88.5%–96.2% in milk. Wang et al. (2021c) developed a bacteria-imprinted conductive poly (3-thiopheneacetic acid) (BICP) film-based impedimetric sensor for the rapid, sensitive, and label-free detection of *S. aureus*.

## Biosensor detection technology

Biosensor is an analysis device consisting of a biosensor and transducer. It mainly uses antigens (antibodies), various sensitive enzymes, alkaloids, and gene sequences to detect microorganisms (Shiba 2006; Wang et al., 2013; Liu et al., 2017c; Ma et al., 2020b; Guo et al., 2021; Das and Mishra, 2022). When the sample to be tested reacts with the above substances, biological interactions will occur, which can then be converted into measurable electrical signals by signal transducers, which can be read and detected by signal amplifiers (Lai et al., 2018a; Lai et al., 2018b; Huang et al., 2021). According to different working principles, biosensors can be divided into optical biosensors, electrochemical biosensors, enzyme biosensors, physical biosensors, mechanical biosensors, and so on (Liu et al., 2018; Liu et al., 2019d; He et al., 2019; Ahovan et al., 2020; Naresh and Lee, 2021; Yu et al., 2021). Biosensors commonly used for rapid detection of foodborne pathogens include; optical biosensors and electrochemical biosensors (Tian et al., 2019; Wu et al., 2020b; Du et al., 2020b; Magesa et al., 2020; Mei et al., 2022).

Optical biosensors are widely used in the detection of foodborne pathogens due to their rapid detection and high sensitivity (Nie et al., 2014; Sun et al., 2021a; Wei et al., 2021). At present, the main optical sensing technologies include chemiluminescence, colorimetry, fluorescence, and surface plasmon resonance (Luan et al., 2020).

**FIGURE 5:**
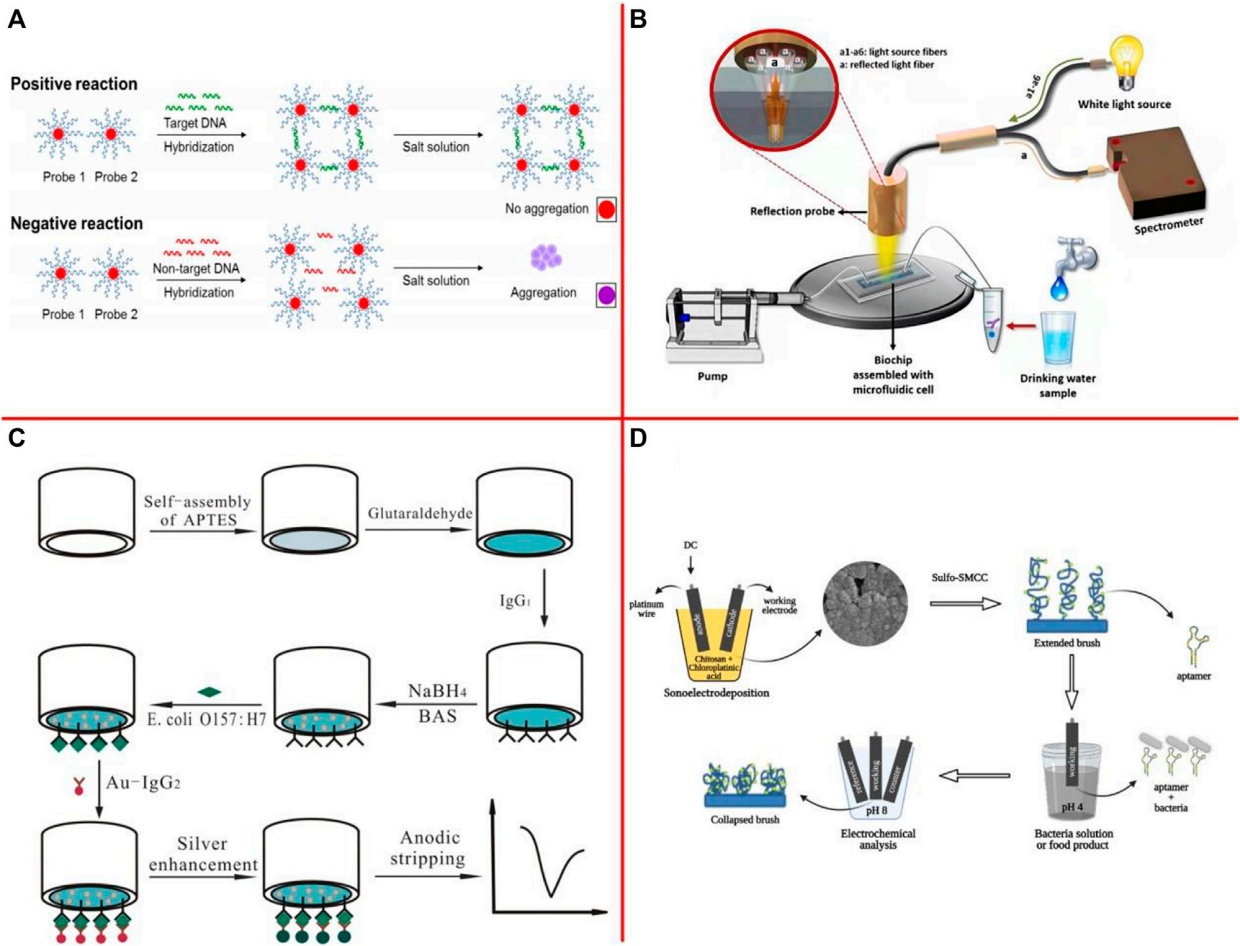
**(A)** The novel principles behind AuNPs optical biosensing and the schematic representation of DNA sandwich hybridization targeting ttrRSBCA locus of *Salmonella* spp. (Quintela et al., 2019); **(B)** Illustration of the white light reflectance spectroscopy (WLRS) optical setup and sensing and sensing principle (Angelopoulou et al., 2021); **(C)** Schematic diagram of electrochemical detection of *E. coli* O157:H7 by gold nanoparticle-catalyzed silver enhancement at porous pseudo-carbon paste electrode (Xu et al., 2012); **(D)** Fabrication, biofunctionalization, and sensing scheme of CHI/Pt aptasensor (Oliveira et al., 2021).

Quintela et al. (2019) developed a novel approach for simultaneous optical detection of various *Salmonella* spp. strains in contaminated complex matrices by utilizing oligonucleotide-functionalized AuNPs as a sensitive optical biosensing platform in combination with an efficient sample pooling and IMS system that ensure the detection of viable cells (Figure 5A). The results showed that the highly sensitive assay toward its target with a superior detection limit of <10 CFU/ml or g and 100% specificity. Srivastava et al. (2021) developed a nanophotonic structure with electric control-based photocatalytic nanocomposite to realize label-free optical detection of foodborne pathogens. The fabricated biosensor is capable of detecting *E. coli* bacteria concentrations of 5,000 CFU/ml. Angelopoulou et al. (2021) presented an optical biosensor for the detection of *S*. *typhimurium* lipopolysaccharide (LPS) and *Salmonella* bacteria in drinking water, based on white light reflectance spectroscopy (Figure 5B). The total assay duration was 15 min, while the achieved detection limits were 4 ng/ml for LPS and 320 CFU/ml for bacteria.

The electrochemical biosensor uses the electrode as a signal converter, and the target analyte performs an electrochemical reaction on the electrode interface, which causes the change of current, potential, impedance, or conductivity on the sensor surface (Deng et al., 2013a; Deng et al., 2013b; Upasham et al., 2021; Vidic and Manzano, 2021). Xu et al. (2012) described the fabrication of three different electrodes based on functional porous pseudo-carbon paste electrodes (PPCPEs) (Figure 5C). A linear relationship between the anodic stripping peak current and the concentration of *E. coli* O157:H7 from 1.0 × 10^3^ to 1.0 × 10^7^ cells/ml and a limit of detection as low as 8.0 × 10^2^ cells/ml were obtained when PPCPE-CHO was used. The target analyte can be quantified by monitoring the change of these signals. Oliveira et al. (2021) developed a label-free and rapid electrochemical biosensor for LM detection using a new one-step simultaneous sonoelectrodeposition of platinum and chitosan (CHI/Pt) to create a biomimetic nanostructure that actuates under pH changes (Figure 5D). Actuation led to improved LM detection with a low limit of detection (33 CFU/10 ml in chicken broth). Feng et al. (2022b) constructed an electrochemical immunosensor for *Salmonella* detection by using a Fe_3_O_4_@graphene modified electrode. Under optimized experimental conditions, a good linear relationship was achieved in the *Salmonella* concentration range of 2.4 × 10^2^ to 2.4 × 10^7^ CFU/ml, and the limit of detection for the immunosensor was 2.4 × 10^2^ CFU/ml.

Biosensor technology detection of microorganisms has rapid, sensitive, simple operation and low requirement for operating personnel, but biological sensors are used to identify biological molecules of the original life is relatively short, with high-cost production, so the use of biosensor technology is limited by some, and most is still in the development stage (Shen et al., 2021c).

## Mass spectrometry

With the development of mass spectrometry, mass spectrometry (MS) is a new detection method of pathogenic bacteria, that has been developed gradually (Fang et al., 2021b). MS is a non-biochemical instrumental analysis method, which takes the characteristics of bacteria or their proteins as the research object and realizes the identification and detection of target bacteria by analyzing the characteristic ions generated after ionization.

Feucherolles et al. (2022) combined Matrix-assisted laser desorption/ionization time of flight mass spectrometry (MALDI-TOF MS) protein mass spectra with a prediction approach as an antimicrobial resistance (AMR) screening tool for relevant foodborne pathogens, such as *Campylobacter coli* and *C. jejuni*. A maximum sensitivity and precision of 92.3% and 81.2%, respectively, were reached. Moreover, Li et al. (2022) presented a novel strategy using mass tag-mediated surface engineering for simultaneous detection of multiple bacteria by MALDI-TOF MS. This strategy converted the detection of bacteria to the analysis of mass tags, allowing simultaneous detection of multiple bacteria and avoiding the dependence of microbial mass spectra databases. Dias et al. (2022) used gas chromatography-mass spectrometry (GC-MS) and gas chromatography-flame ionization detector (GC-FID) to determine the antibacterial activity of three essential oils (EOs) and their main components against foodborne pathogens and spoilage foods.

Among many detection technologies, the foodborne pathogenic bacteria MS has the highest detection rate at present, with fast detection speed and convenient operation, but there are still many problems in the actual detection process (Mangmee et al., 2020). In the detection process, it is necessary to improve the sensitivity and stability of foodborne pathogenic bacteria MS technology, so it is necessary to constantly debug spray voltage, flow rate, capillary temperature, and other issues to achieve the optimal state.

## Conclusion

In recent years, many methods for the detection of foodborne pathogens have been developed to address food safety and public health issues, especially with the increased consumption of fresh food and food with short shelf life. Rapid detection technologies are becoming more marketable (Freitas et al., 2020; Ezzatpanah et al., 2022). For example, many scholars have combined POCT technologies such as molecular immunology, bio-molecular, biosensor, and microfluidics to provide new approaches for rapid, low-cost, highly sensitive, and highly specific detection methods for foodborne pathogens (Cimafonte et al., 2020; Wang et al., 2021d; Zhang et al., 2021c). The POCT technology provides simple, fast, and sensitive platforms for the detection of foodborne pathogens and will become a powerful multi-functional tool for food safety, biological threat detection, and environmental monitoring. However, there are still shortcomings that require researchers to continuously improve the existing detection technologies (He et al., 2021b; Wagner et al., 2021). The sensitivity, specificity, ease of operation and detection speed, cost, and application of the techniques for detecting foodborne pathogens are given in Table 1.

**TABLE 1:** Compare the methods of detecting foodborne pathogens.

Detection technology	Sensitivity	Specificity	Operation	Speed	Cost	Application	References
Immunology	202 CFU/ml	High	Easy to use	12 min	—	*C. sakazakii*	Gao et al. (2021)
	10 CFU/ml	High	Professionals to operate	65 min	—	*S. aureus*	Yao et al. (2020)
	100 CFU/ml	Extremely good	Professionals to operate	150 min	—	*V. parahaemolyticus*	Zhai et al. (2021)
	2 CFU/g	High	Easier to operate	4 h	—	*V. parahaemolyticus*	Jiang et al. (2020)
	10^4^ CFU/ml	High	Easy to use	<3 h	Less costly than C-ELISA	*E. coli* O157:H7	Zhao et al. (2020)
	6 CFU/ml	Good	Professionals to operate	10 h	—	*S. enteritidis*	He et al. (2020)
Molecular	940 CFU/g	High	Professionals to operate	45 min	—	*L. monocytogenes*	Li et al. (2021b)
	1 CFU/ml	>80%	Professionals to operate	8 h	—	*Salmonella*	Du et al. (2021b)
	540 CFU/ml	Very high	Almost equipment-free	70 min	Low cost	*S. aureus*	Zhou et al. (2022)
	10 CFU/ml	Good	The whole operational procedure should be finished coherently	30 min	—	*S. typhimurium*, *Brucella* spp., *B. cereus* and *Shigella* spp.	Shi et al. (2019)
Microfluidics	15 CFU/ml	Good	Semi-automatic operation	about 1 h	—	*S. aureus*	Song et al. (2020)
	5.74 CFU/ml	High	Professionals to operate	about 30 min	—	*V. parahaemolyticus*	Jiang et al. (2021)
	44 CFU/ml	Good	Semi-automatic operation	45 min	—	*Salmonella*	Xue et al. (2021)
	0.5 CFU/ml	Good	Professionals to operate	about 1 h	A very low number of antibodies are needed	*E. coli* O157:H7	Asgari et al. (2022)
Metabolic	2 CFU/ml	High	Professionals to operate	10 min	Production cost of the BICP film was low	*S. aureus*	Wang et al. (2021c)
	80 CFU/ml	Good	Professionals to operate	2 h	—	*Salmonella*	Wang et al. (2020g)
	In water and milk are 7.1 and 9.2 CFU/ml	Good	Professionals to operate	within 1 h	—	*L. monocytogenes*	Wang et al. (2021b)
Biosensor	320 CFU/ml	High	Professionals to operate	15 min	The WLRS Biochip can be reused to reduce costs	*Salmonella*	Angelopoulou et al. (2021)
	240 CFU/ml	Excellent selectivity	Professionals to operate	1.5 h	—	*Salmonella*	Feng et al. (2022b)
	5,000 CFU/ml	Good	Professionals to operate	1 min	—	*E. coli*	Srivastava et al. (2021)
	<10 CFU/ml or g	High	Professionals to operate	<1 h	—	Various *Salmonella* spp	Quintela et al. (2019)
Mass Spectrometry	99.3% accuracy	High	Not require specially trained staff	The analysis time is longer than immunoassays and nucleic acid-based assays	Low cost per sample, but high initial cost of the instrument	Non-typhoidal *Salmonella* serovar screening	Mangmee et al. (2020)
	*S. aureus* (1,000 CFU/ml), E. coliO157:H7 (500 CFU/ml)	High	Professionals to operate	—	—	*E. coli* O157:H7 and *S. aureus*	Li et al. (2022)

*C. sakazakii*, *Cronobacter sakazakii*; *V. parahaemolyticus*, *Vibrio parahaemolyticus*; *S. aureus*, *Staphylococcus aureus*; *S. enteritidis*, *Salmonella enteritidis*; *S. Typhimurium*, *Salmonella Typhimurium*; *B. cereus*, *Bacillus. cereus*; *E.coli*, *Escherichia coli*; *E. coli* O157:H7, *Escherichia coli* O157:H7; C-ELISA, conventional enzyme-linked immunosorbent assay; BICP, bacteria-imprinted conductive poly; WLRS, white light reflectance spectroscopy.

With the advancement of science and technology, artificial intelligence, gene editing, nanotechnology, and other cutting-edge disciplines, the integration of these technologies and POCT technology in the rapid detection of foodborne pathogens will also become the future development trend (He et al., 2018; Chen et al., 2020c; Ding et al., 2020; Peng et al., 2020b; Yang et al., 2020c; Gong et al., 2021; Xiao et al., 2022). The following is the prospect of POCT technology in the future development trend: 1) the detection index is gradually transformed from biochemical and immunity to nucleic acid molecules, and from single to multiple indicators. 2) The devices are more miniaturized and integrated by the development of micro-nano fabrication and 3D printing and new materials technology. More and more functions can be integrated into a small device, such as integrate sample extraction and detection into a chip our cassette, etc. 3) The devices have the features of higher sensitivity and quicker time with the application of novel CRISPR method and gold nanoparticles and so on. 4) The devices will be more convenient and lower cost for the use of smart mobile phones, lateral flow dipsticks, or paper chips. Moreover, with the advance of cloud computing, Internet of things technology, devices make more intelligence.

The future detection technology for foodborne pathogenic bacteria will focus toward the integration of a variety of detection technologies, making flux detection faster, with higher sensitivity and repeatability, faster time, and lower cost. The future will also include wide promotion and standardization direction for these technologies, including production, processing, distribution, and sale of the whole production chain of food safety regulations.
